# Current Concepts in Shoulder Periprosthetic Joint Infections—Are Shoulders the Same as Hips and Knees?

**DOI:** 10.3390/jcm14082578

**Published:** 2025-04-09

**Authors:** Florian August Frank, Andreas Marc Müller, Mario Morgenstern, Richard Kuehl, Martin Clauss

**Affiliations:** 1 Center for Musculoskelettal Infections (ZMSI), University Hospital Basel, 4031 Basel, Switzerland; florian.frank@usb.ch (F.A.F.); mario.morgenstern@usb.ch (M.M.); richardalexander.kuehl@usb.ch (R.K.); 2 Department of Orthopaedic Surgery and Traumatology, University Hospital Basel, 4031 Basel, Switzerland; a.mueller@usb.ch; 3 Department of Infectious Diseases, University Hospital Basel, 4031 Basel, Switzerland

**Keywords:** periprosthetic joint infection, shoulder arthroplasty, narrative review, current concepts

## Abstract

**Background/Objectives**: The vast amount of research and data on periprosthetic joint infection (PJI) is focussed on infections in hip and knee replacements. This article aims to highlight the special features of PJI in shoulders. **Methods**: This narrative review is based on the recent and most relevant literature regarding PJI in general, and in shoulders in particular. **Results**: While the majority of findings for PJI in hips and knees can be transferred to infected shoulder arthroplasties, shoulder PJI represents a unique entity with a different microbial profile and its own diagnostic challenges. **Conclusions**: As profound evidence for shoulder PJI is lacking, diagnostic and therapeutic algorithms should be transferred from those for PJI in hips and knees. Further research is necessary to determine optimal management of shoulder PJI.

## 1. Introduction

Most classifications and treatment algorithms for periprosthetic joint infection (PJI) were developed based on data and experience regarding PJI in knees and hips; their applicability to shoulder PJI is unknown.

PJI occurs in approximately 1% of primary and about 10% of revision shoulder joint replacements [1,2]. Infections are the main cause for revision in painful shoulder arthroplasties [3]. Portillo and colleagues were able to show, in a cohort consisting mainly of hip and knee replacements, that any revision within the first two years after implantation has a high probability of being infected [4]. Shoulder PJIs pose a challenge, as they usually present less symptomatically as low-grade infections, and *Cutibacterium acnes* can be cultivated in up to 70% of cases [3,5,6]. The microbial profile in shoulder PJI differs compared to hip and knee PJI. While the rate of Gram-negative organisms is very similar, the distribution of coagulase-negative *staphylococci* and *Cutibacterium acnes* are reversed (Table 1).

The following narrative review is intended to shed some light on shoulder PJI in the context of current classifications and definitions of, and diagnostic and treatment strategies for, PJI.

## 2. Classifications

Several classifications have been proposed to help diagnose PJI. One of the earliest and still regularly applied classifications is the Centers for Disease Control and Prevention (CDC) guideline on surgical site infections (SSIs) based on a publication by Horan et al. in 1992 [10]. As its name suggests, this classification covers every surgical site infection and is not tailored to PJI in particular. Its biggest drawback, however, is that differentiation, if a deep or superficial infection is present, is made by the surgeon based on subjective diagnostic criteria. Thus, it lacks objectivity [10].

**Figure 1:**
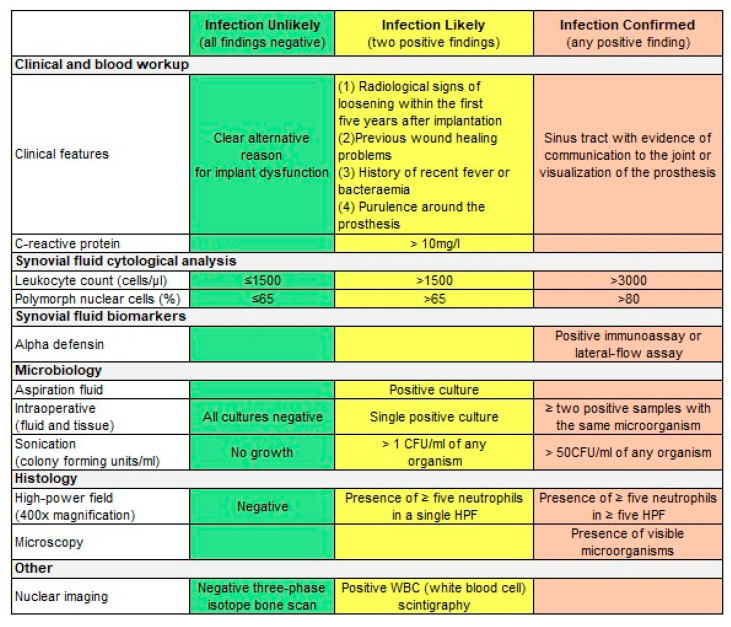
In accordance with the EBJIS definition of PJI [15].

In 2011, the Musculoskeletal Infection Society (MSIS) published the first definition specifically designed for PJI [11], which was modified in 2013 and presented at the International Consensus on Musculoskeletal Infection (ICM) [12]. In the same year (2013), the Infectious Disease Society of America (IDSA) [13] followed with diagnostic criteria defined by an expert panel. In 2018, an attempt was made to include an “uncertain” group into the MSIS criteria during yet another ICM meeting in Philadelphia. The MSIS did not adopt those latest suggestions [14]. These three definitions (original MSIS, IDSA and ICM-modified MSIS) include a passage stating that infection may be present even if the defining criteria are not met. In 2021, the European Bone and Joint Infection Society (EBJIS) released a PJI classification that defined three different groups according to the likelihood of infection—infection unlikely, likely and confirmed [15] (Figure 1).

To date, the modified ICM criteria of 2018 remain the most often used definition in research. Most diagnostic tests are tuned to this definition, and test performance is measured using these criteria. Any change in the underlying definition changes sensitivity and specificity [16].

Recent studies were able to show that the EBJIS definition had the best sensitivity in identifying PJIs in hips and knees and produced the smallest percentage of uncertain results [17]. The rate of correct preoperative diagnosis of PJI was also highest with the EBJIS definition [16,17]. This is exceptionally important in low-grade infections, as underdiagnosing infection could lead to inferior outcomes due to inappropriate or delayed treatment [17]. Despite these differences in the sensitivity of diagnosing infection, Sousa et al. demonstrated that all classifications achieved comparable outcomes in terms of infection-free survival [17]. In the following sections, we discuss the applicability of these diagnostic criteria to shoulder PJI.

## 3. Diagnostics

### 3.1. Clinical Findings

Classical clinical findings suggestive of an early postoperative infection are wound healing abnormalities and loosening of the implant.

Wound healing impairment is a common problem in the postoperative period that has been reported in up to 10% of patients after primary total knee arthroplasty (TKA) [18]. As most postoperative wounds are managed by patients at home, their general practitioner or community nurses, wound healing problems may be underreported.

Persistent pain and stiffness are typical signs of a PJI, especially following shoulder arthroplasty. Any loosening of the humeral stem has to be considered an infection until proven otherwise [5]. Early prosthetic loosening is mostly due to mechanical failure or infection [4]. The later the failure, the less likely it is due to an infection, and the more likely it derived from aseptic loosening. A total of 50% of septic failures occur within the first 2 years, and 90% within the first 5 years, after surgery [4]. We therefore recommend that in all revisions performed for loosening within 2 years of implantation, PJI should be considered, even in the absence of clinical or laboratory findings suggestive of infection. This is particularly true if the humeral stem shows signs of early loosening.

### 3.2. Joint Aspiration

Joint aspiration is one cornerstone of diagnosing PJI. Aspiration can detect the body’s immune response in the synovial fluid (via white blood cell count and cell differentiation) and helps identify the causative pathogen as well as possible crystal deposits.

Gram staining of synovial fluid has a low sensitivity of approximately 20%. Thus, it should not be performed as a routine test to rule out infection. If, however, bacteria are identified via Gram staining, the diagnosis of infection is confirmed [19].

White blood cell (WBC) count and the percentage of polymorph nuclear cells (PMNs) both have a sensitivity and specificity of around 90% with the above-mentioned cut-off values for PJI (Figure 1) [20,21,22]. Levent et al. showed that synovial cell differentiation and leukocyte count had the best performance in diagnosing PJI according to ICM criteria [23]. However, in infections with *C. acnes*, synovial fluid analysis may render unreliable results [24,25].

Only a few studies have specifically investigated cut-offs for cell count and cell differentiation in shoulder PJI (Table 2). The ICM classification was used twice for reference [26,27], as was the IDSA classification [28,29]. One study tested all available definitions [30]. Their pooled results (WBC cut-off of 700–12,000 and PMNs > 54%) suggest that the EBJIS cut-offs for leukocytes and polymorph neutrophiles [15] are also valid for PJI in shoulders.

Microbiological cultures from joint aspirates turn positive in approximately 60–80% (depending on the microorganism) of cases with confirmed periprosthetic infections [31,32]. The use of paediatric blood culture bottles aids in achieving better results with a sensitivity of 91% [33].

### 3.3. Intraoperative Sampling

Superficial swabs should be avoided, as they carry a high risk of contamination and their sensitivity for detecting the causative pathogen is low [34]. Samples obtained from the sinus tract are prone to colonisation from the surrounding skin, which may or may not be part of the infectious microbiome [35].

Intraoperative sampling is the gold standard to diagnose PJI and the causative pathogen. To standardise diagnostics, intraoperative sampling should ideally follow a pre-defined protocol [36]. Rather than splitting one large piece of tissue into multiple samples, at least five separate samples should be taken at different locations adjacent to the implant to ensure the causative pathogen is detected and identified. Each sample should be divided and sent for histopathology [37] and microbiology [38,39], with corresponding samples being labelled in the same way. Implants should be sent for sonication [40].

Samples with suspected low-grade infections should undergo prolonged incubation, as anaerobic organisms have been shown to grow later than aerobic pathogens, with a relevant proportion growing after day seven [41]. Anaerobic handling in the microbiology laboratory can help expedite the detection of anaerobes and other slow-growing organisms [42], but is not available in every laboratory.

After receiving definite results, microbiological and histological samples can be matched to discriminate between “true infection” and “contamination”. This is particularly important in suspected shoulder PJIs with limited samples growing *Cutibacterium acnes* with uncertain significance (Table 3).

One recent review focussing on diagnostic arthroscopy for sampling in shoulder PJI found only limited low-level supportive evidence [43]. If arthroscopic samples are taken, these should derive from bony specimens as close as possible to the implant, as synovial biopsies have been shown to produce a high rate of false positive results for *Cutibacterium acnes* [44,45].

### 3.4. Advanced Diagnostics

As a polymerase chain reaction (PCR) can identify bacterial DNA, it is helpful to not only confirm PJI, but also identify the causative pathogen—especially in pre-treated patients, where pathogens may not grow anymore in routine culture. One drawback of PCR is that antibiotic susceptibility testing is not possible. Recently, multi-panel kits were developed to expedite pathogen identification. Their reported sensitivities and specificities are 90% and 99%, respectively. Only a small sample of synovial fluid (0.2 µL) is needed, and results are available within one hour. One should be aware that some currently available multi-panel kits do not include *Coagulase negative Staphylococci* and *Cutibacterium acnes*, the two most relevant low-grade organisms, because of concerns regarding overreporting PJI due to common contamination [46].

Next generation sequencing (NGS) is a relatively new method for detecting bacterial DNA and/or RNA. As fragments of genetic information in a given sample are sequenced and amplified simultaneously, a complete microbial profile is generated. A recent review by Hantouly et al. showed very good results that may even be superior to conventional cultures in the diagnosis of PJI [47]. Data on the value of NGS in diagnostics for total shoulder arthroplasty in particular are unavailable.

### 3.5. Biomarkers

Biomarkers have their highest value in detecting PJI in low-grade infections, as PJIs with highly virulent pathogens like *S. aureus* or *E. coli* present with clear clinical signs of infection and can usually be detected by conventional cultures. Biomarkers may help interpret positive cultures in low-grade infections and differentiate true infection from contamination. To help with this challenge, the interest in biomarkers assisting in diagnosing PJI is high. The biomarker commonly being tested, C-reactive protein (CRP), is not a good marker to identify infection, especially low-grade infections [48,49]. Carli et al. conducted a review in 2019 that identified 203 studies on 83 different biomarkers or tests. Most studies (~85%) were prone to a high risk of bias, meaning only 33 studies did not have a high risk of bias [50].

Similarly, Sigmund et al. showed that any biomarker available, long-established or new to the market, had inferior diagnostic performance for joint aspiration and clinical examination [51]. These general findings regarding biomarkers in PJI can be transferred to shoulder PJI, as most tests provide similar results for PJI in general and shoulder PJIs [52,53,54].

### 3.6. Summary

Clinical suspicion and synovial fluid work-up still serve as major pillars in diagnosing PJI. The sensitivity of microbiological diagnostics has improved over the years, and NGS might be a promising tool. Thus far, NGS is not included in any classification system. Current biomarkers are helpful in ruling out PJI, but they substantially raise the cost in the diagnostic algorithm, which is especially challenging in times of limited money and resources in most healthcare systems. As biomarkers show limited additional benefit in diagnosing low-grade infections, hopes are high for next generation sequencing.

## 4. Treatment Strategy

**Figure 2:**
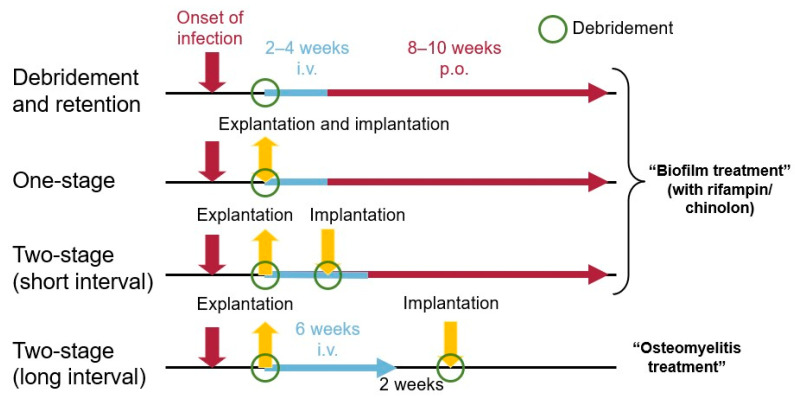
Treatment concept of Zimmerli, Trampuz and Ochsner [55] published in Mandell, Douglas and Bennett [61].

There are various options for the surgical treatment of PJI (DAIR, one-stage and two-stage procedures) with substantial differences in underlying treatment concepts, as published by Zimmerli et al. in 2004 [55]. Taking a closer look at these options, there are fundamental differences in the underlying treatment philosophies. Two-stage revisions with a long interval (normally > 6 weeks) represent an osteomyelitis treatment. The biofilm located on the foreign material (implant, bone cement or dead bone sequestra) is surgically removed and surgery is succeeded by antibiotic treatment for a minimum of 6 weeks [56]. After curing the osteomyelitis, the new implant is placed into a “sterile” bone bed. In contrast, during DAIR and one-stage procedures residues of the biofilm remain on the implant (DAIR) or a new biofilm starts to form immediately after the new implant is placed in the still infected bone bed (single-stage exchanges). In the less frequently used but well established two-stage exchange with a short interval (2–4 weeks between stages), the soft tissue infection is cured [57], but the osteomyelitis is still active, thus a new implant is expected to be colonised by biofilm-forming bacteria. Therefore, postoperative biofilm-active antibiotic treatment is mandatory for treatment success in these cases (Figure 2). The only significant change to the concept proposed by Zimmerli et al. [55] in the last 20 years is the duration of intravenous antibiotics, which has been cut short due to studies showing the non-inferiority of oral versus intravenous antibiotics in osteoarticular infections [58,59]. Those results have recently been supported by a study focussing particularly on periprosthetic joint infections [60].

For infected shoulder arthroplasties, recent publications have found comparable infection eradication for single-stage vs. two-stage revisions, with a tendency toward benefit in terms of functional outcome in single-stage revisions [62,63,64]. This may be due to a high percentage of infections with well-treatable pathogens (*Cutibacterium acnes*). DAIR procedures showed similar reinfection rates and similar patient-reported outcomes compared to “exchange” procedures [65]. Furthermore, compared to PJI after THA/TKA, it is noteworthy that during two-stage revisions of shoulder PJIs, about one in three patients opted not to have the second-stage surgery performed [66].

## 5. Setting

It is well established for the treatment of bone and joint infections, and PJI in particular, that outcomes are superior when patients are treated by a multidisciplinary team (MDT) [67,68,69]. These teams should consist of orthopaedic surgeons, infectious disease and microbiology specialists, specialised nurses and plastic surgeons. Not to be forgotten, social, psychological and nutritional support team members are of utmost importance to achieve successful outcomes [70].

## 6. Conclusions

As profound knowledge is lacking, diagnostic and therapeutic algorithms for shoulder PJI should be transferred from those for PJI in hips and knees. Clinical awareness/observation and joint aspiration are the two most important cornerstones for diagnosing PJI in shoulders. Thresholds for cell count and cell differentiation are within the same range as for PJI in knees and hips.

One-stage exchanges are the treatment of choice, as they show better results in terms of functionality. We strongly advocate treating these patients as a multidisciplinary team to ensure optimal outcomes.

As abundant evidence on shoulder PJI is still lacking and multiple questions are unanswered or answered by assumption, further research is necessary to determine optimal diagnosis and treatment.

## Figures and Tables

**Table 1:** Common pathogens identified in PJI.

	PJI Hips/Knees			PJI Shoulders		
	Fröschen et al. [7]	Li et al. [8]	Tai et al. [9]	Marigi et al. [6]	Richards et al. [5]	Pottinger et al. [3]
Coagulase-negative *staphyloccoci*	49%	51%	37%	13%	14%	n/a
*S. aureus*	13%	7%	24%	19%	14%	n/a
*E. faecalis*	7%	4%	8%	1%	6%	n/a
Gram-negative bacteria	14%	12%	11%	13%	16%	n/a
*C. acnes*	4%	0%	8%	44%	31%	69%

**Table 2:** Proposed cut-off values for synovial fluid in shoulder PJI.

Author	Number of Patients	Year	WBC (/µL)	Sens/Spec	PMNs (%)	Sens/Spec	Definition Used
Strahm C. et al. [29]	19	2018	>12,200	92/100	>54	100/75	IDSA
Huard M. et al. [30]	136	2020	>3077	91/85			several
Patel V.V. et al. [26]	87	2021	>3000	30/100	>80	20/100	ICM
Streck L.E. et al. [28]	31	2021	>2800	87/88			IDSA
Streck L.E. et al. [27]	35	2022	>700	86/100			ICM

WBC = white blood cell count; Sens = sensitivity; Spec = specificity; PMNs = polymorph nuclear cells.

**Table 3:** Interpretation of microbiology and histopathology from correlating intraoperative samples.

Sample	Microbiology	Histopathology	Judgement
1	Low ^a^ or high virulence ^b^ pathogen(s)	Inflammation	Infection
2	Low virulence pathogen(s) ^a^	No inflammation	Contamination/Colonisation
3	High virulence pathogen(s) ^b^	No inflammation	Likely infection
4	No growth	No inflammation	No Infection
5	No growth	Inflammation	Infection possible ^c^

^a^ e.g., *Cutibacterium acnes*, *CoNS*; ^b^ e.g., *S. aureus*, *E. coli*, other Enterobacterales; ^c^ consider diagnostics for culture-negative infection (NGS/PCR) or alternative non-infectious etiology.

## Abbreviations

The following abbreviations are used in this manuscript:

CoNS	Coagulase-negative staphylococci;
CRP	C-reactive protein;
DAIR	Debridement, antibiotics and implant retention;
DNA	Deoxyribonucleic acid;
EBJIS	European Bone and Joint Infection Society;
ICM	International Consensus on Musculoskeletal Infection;
IDSA	Infectious Disease Society of America;
MDT	Multi-disciplinary team;
MSIS	Musculoskeletal Infection Society;
PCR	Polymerase chain reaction;
PJI	Periprosthetic joint infection;
PMN	Polymorph nuclear cell;
THA	Total hip arthroplasty;
TKA	Total knee arthroplasty;
WBC	White blood cell.
